# Supplementary material for: The microbiome biomarkers of pregnant women’s vaginal area predict preterm prelabor rupture in Western China
Source: Front Cell Infect Microbiol. 2024 Oct 31;14:1471027. doi: 10.3389/fcimb.2024.1471027 (PMC11560878; doi:10.3389/fcimb.2024.1471027)
Supplement: Supplementary file 1 [file DataSheet1.zip › compare_1/Community/Pieplot/order/group/C.order.pieplot.pdf]

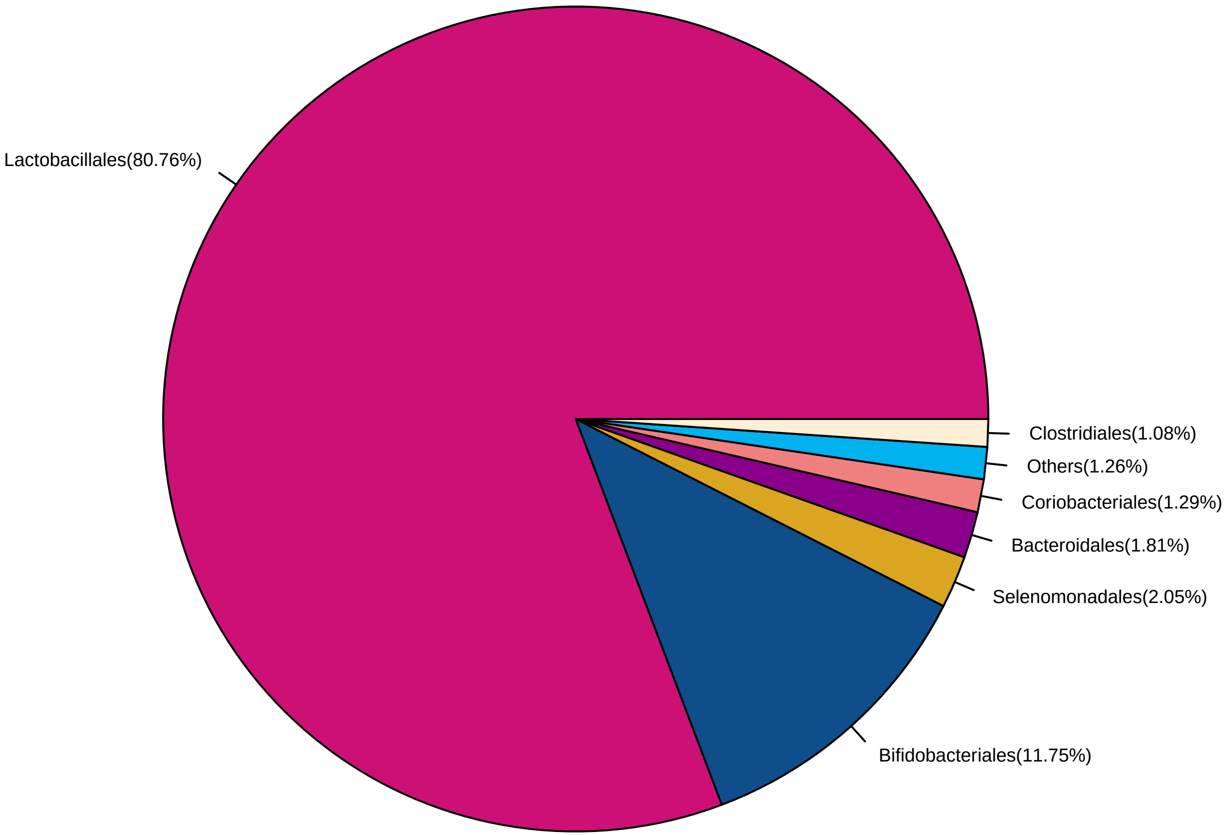
Lactobacillales(80.76%)
Clostridiales(1.08%)
Others(1.26%)
Coriobacteriales(1.29%)
Bacteroidales(1.81%)
Selenomonadales(2.05%)
Bifidobacteriales(11.75%)
